# COVID-19, 5G conspiracies and infrastructural futures

**DOI:** 10.1177/1329878X20952165

**Published:** 2020-11

**Authors:** James Meese, Jordan Frith, Rowan Wilken

**Affiliations:** RMIT University, Australia; Clemson University, USA; RMIT University, Australia

**Keywords:** 5G, conspiracy theories, COVID-19, economic statecraft, mobile infrastructure

## Abstract

This article examines the emergence of conspiracy theories linking COVID-19 with 5G, with a focus on Australia, the United States and United Kingdom. The article is in two parts. The first details long-standing concerns around mobile technologies and infrastructures before showing how they translate to specific worries about 5G technology. The second shows how these fears have fuelled specific conspiracies connecting 5G with COVID-19, how they have animated protests and acts of vandalism that have occurred during the pandemic, and the ongoing engagement of conspiracists with official inquiries into 5G. Finally, we argue that a productive way to understand what is happening with 5G is to look beyond conspiracy theories to a larger set of concerns. We argue that the battle for control of 5G infrastructure can be productively understood in geopolitical terms, as forms of economic statecraft, which partly explains why governments are increasingly concerned about countering misinformation and disinformation around 5G.

## Introduction

One of the more unexpected events to take place during Australia’s early wave of COVID-19 restrictions was a series of public protests staged in Melbourne and Sydney on 11 May 2020, and again (including Brisbane) on May 30 (Brown and Loomes, 2020; TND, 2020). While ostensibly organised in protest of COVID-19 lockdown measures, those present also bore anti-vaccination and anti-5G signs and placards. A month earlier, on 22 April, there had been a rally in the northern NSW town of Mullumbimby protesting the rollout there of 5G infrastructure by telecommunications provider Telstra (Bibby, 2020) – a protest to which we shall return later in this article. However, what set the subsequent capital city protests apart from this one was the unexpected bundling up of 5G with COVID-19-related complaints. These protest events were reported at the time as minor events organised by conspiracy theorists and other fringe elements. With hindsight, they can now be regarded as early, visible signs of what were to become much more widely circulated concerns and coordinated campaigns that sought to link COVID-19 and 5G technology.

In this article, we draw on news reports and other published sources and bring together Australian, US and UK examples to examine how this connection between COVID-19 and 5G has come about, how it can be situated within a longer history of concern around mobile technologies, and its impacts on policy preparation for and response to the rollout of 5G infrastructure. The article is structured in two parts. In the first part, a backdrop to these 5G-COVID-19 concerns is painted by providing a brief history of concern around mobile technologies and infrastructures, explaining what 5G technology is and what it entails, and examining concerns around electromagnetic field (EMF) exposure and 5G. Against this backdrop, in the second part of the article, we explore the conspiracy theories linking 5G and COVID-19, and how these concerns have played out on the ground, paying particular attention to how conspiracists have engaged with government inquiries into 5G. We conclude by considering some of the larger political economic and geopolitical factors shaping debates around 5G, and we reflect on some of the broader implications of these campaigns for 5G rollout.

## Part I: context

### Mobile and communication infrastructure: fears and concerns

Green Bank, West Virginia, is a small town in the Appalachian Mountains of the United States. According to the 2010 census, the town had about 150 people living there, and no real city to speak of. One could drive right through Green Bank and barely notice it, except for one thing: mobile phones will not receive signal. If those passing through stop and scan for Wi-Fi, they will not find a signal. This is because Wi-Fi and cell signals are illegal. One might even struggle to find a house with a microwave. In fact, there are almost no radio transmissions at all within the town limits, a restriction mandated by Federal Law because Green Bank is part of the 33,669 km^2^ (13,000 mile^2^) National Quiet Zone that restricts wireless signal and makes the restrictions more severe the closer you get to Green Bank. Green Bank itself also bans pretty much any kind of radio signal because it is the home to the Green Bank Observatory that features the world’s largest steerable telescope. One of the Observatory’s goals is to scan for distant signals, which is why Green Bank restricts wireless signals; a cell signal is just too loud when listening for signals from millions of miles away.

For most people, Green Bank, WV, is a curiosity, a small rural town free from the radio signals that have shaped contemporary life. But, for a small group of people, Green Bank represented hope of a ‘healthier’ future. Throughout the 2000s and 2010s, a ‘small, but significant population’ of people – described by the BBC as ‘electro-sensitive refugees’ – have moved to Green Bank specifically to avoid wireless signals (Holba and Hall, 2015: n.p.). These people had long felt that wireless signals, from both Wi-Fi and cellular networks, were making them sick. They described symptoms ranging from fatigue to hair loss and linked those symptoms to the invisible radio signals all around us. Or, to put it differently, they claimed they suffered from ‘electromagnetic sensitivity’, despite it not being a recognised medical diagnosis. Most of those people obviously cannot flee to radio-free towns like Green Bank.

Electromagnetic sensitivity is far from the only affliction people claim is caused by wireless signals. In the early 2000s, there was significant public debate about Wi-Fi, cell tower base stations and so forth and links to various types of cancer (Sutherland, 2007). Many popular press books of questionable quality have been devoted to the dangers of EMF exposure, which is described as exposure to electricity of various types. The lists of supposed symptoms of EMF exposure are too long to list here in full. However, in just one book by alternative health guru Joseph Mercola, who is famous for his anti-vaxxer views, EMF exposure from cell phones and Wi-Fi is alleged to be linked to anxiety, infertility, brain cancer, heart disease, breast cancer, attention deficit hyperactivity disorder (ADHD), depression, diabetes and so on (Mercola, 2020). The list goes on and on.

While fears about EMF exposure have flared with the development of 5G, few of these concerns are new. The same fears were expressed in the 1990s and early 2000s, with activists focusing on the dangers of non-ionising radiation. Put simply, EMFs are typically grouped into two types: ionising radiation (like in X-rays) and the non-ionising radiation of power grids and cell phone towers. Ionising radiation has the ability to disrupt atoms at a structural level, while non-ionising radiation does not – a distinction that Mercola (2020: 253) contests. This essentially gets to the crux of the issue. The current view of policy makers is that non-ionising radiation only holds minimal-to-no risk, especially when exposure guidelines put in place by the FCC and other groups are met. Certain fringe health advocates, on the other hand, have argued for more than two decades that this is not the case, suggesting, among other things, that in the United States the standards are too low and that regulatory bodies, like the FCC, are influenced by lobbyists.

This history of concern about mobile networks is important for understanding the COVID-19 conspiracies, even if the specific concerns are different. Past protests of successive generations of mobile networks have already planted the idea that mobile infrastructure has negative health effects, and in some cases these protests have netted results. In the United States in the 1990s and 2000s, for instance, protestors succeeded in changing certain city codes for how close mobile infrastructure could be located to residential areas (Mercola, 2020); they also managed to get some school districts to severely limit Wi-Fi exposure because of similar health concerns (Buck, 2014). Obviously, these protests have not stopped the development of wireless infrastructure, but they have, in certain situations, hindered the rollout and operation of this infrastructure. Similar protests around EMF exposure are then staged again as new generations of mobile standards are introduced, as has happened with the development of 5G. Prior to examining how ongoing concerns about public health and mobile technology have manifested in relation to 5G, it is first worthwhile explaining how 5G technology works and what it is expected to do.

### The 5G revolution

5G operates in a radically different way to previous upgrades to mobile infrastructure. Earlier improvements have generally allowed for faster data transfers, in turn supporting the development of innovative consumer-facing technology. For example, 2G supported the mainstreaming of SMS communication, 3G allowed powerful mobile devices like the iPhone to become a reality and 4G supported streaming media on mobiles and real-time locative technology like Uber. While 5G will help improve consumer experience through faster speeds and lower latency, proponents argue that it will have a much broader impact on society because of how the technology is configured.

5G wireless networks are capable of carrying up to 1000 times the traffic of existing services. In order to accommodate this greater demand, 5G draws on expanded spectrum allocation, utilising higher frequency signals (millimetre waves) that are relayed via hundreds of small cell network receivers or mini base stations. 5G still uses the existing network of larger cell towers, but these will be loaded with hundreds more ports or antennas – technology that is known as ‘massive MIMO’ (multiple input, multiple output devices) – that dramatically increase network traffic capacity (Nordrum et al., 2017). In order to streamline the delivery and flow of all this increased data, 5G also utilises a range of other technologies to send focused streams of data to users (‘beamforming’). This is one of the fundamental changes that differentiates 5G from 4G. While traditional antennae broadcast signals that radiate in all directions, 5G networks will use beamforming to target signals to devices that need them at any given moment. The network will also better manage the two-way flows of data through a signalling or switching system called ‘full duplex’ (Nordrum et al., 2017). Currently, if mobile base stations and mobile phones are operating on the same frequency, they need to take turns when exchanging information or alternatively operate on a different frequency. Full duplex will allow them to ‘transmit and receive data at the same time, on the same frequency’, which would significantly speed up data transfers (Nordrum et al., 2017).

However, MIMO technology will be predominantly located on traditional base stations that previously transmitted 4G. It essentially stands as an improvement on existing technology (Johnson, 2018). The more noticeable change with 5G is that it can also be transmitted through new small cell networks. Small cells are tiny base stations that can provide high-frequency mobile coverage over 200–400 m (656–1312 ft) (Wood, 2019). While they have been used occasionally in previous generations of mobile technology, they will be essential to 5G rollout because the technology has been allocated more high-frequency spectrum, which has lots ‘of capacity and bandwidth [but] over a shorter range’ (Davies, 2019). This is partially due to a lack of available spectrum but also because these higher frequencies can better support the sorts of use-cases that are expected to emerge following the mass deployment of 5G (Davies, 2019).

Small cells are expected to be placed every few hundred metres in densely populated urban areas, providing fast network speeds and low latency resulting in faster response times from the network. These wide-area networks (or WANs) could support an expanded array of technologies and services that rely on WANs, including transport logistics, the Internet of things (IoT), autonomous vehicles and smart city infrastructures. Therefore, the 5G rollout stands as a significant transformation of mobile infrastructure, transforming it into infrastructure oriented at a range of businesses rather than communicative infrastructure oriented at consumers. There are significant economic benefits to be realised if this transformation occurs as planned, with research commissioned by the former Australian Government Department of the Communications and the Arts suggesting that ‘5G networks could contribute up to [. . .] $50 billion in additional GDP’ for Australia (Deloitte, 2018a), and the World Economic Forum anticipating a 13.2 trillion growth in global economic value (WEF-PwC, 2020).

Considering this potential economic windfall and the long history of fears about the development of mobile technology discussed earlier, it is perhaps unsurprising that 5G has become a site of significant contestation. Countries are desperate to roll out this new technology, knowing that rapid deployment could give countries a head-start in establishing new innovations, like an autonomous vehicle network (Wilken and Thomas, 2019), providing a genuine economic boost for the country. This heady atmosphere has led to significant geopolitical tensions around which infrastructure providers should be invited to tender, with Australia excluding Chinese company Huawei from contributing to the build of the national 5G network (Hunter, 2020; Remeikis, 2020). Alongside these political issues, governments have also had to contend with a vociferous group of citizens who argue that 5G poses unacknowledged public health risks, which are being ignored. These arguments are similar to earlier complaints about mobile technology and public health. However, because 5G is a significantly different form of mobile technology, it means that concerns and subsequent protests also manifest in a unique manner, as seen in recent attempts to link the technology to the COVID-19 pandemic. The following section explores how concerns about 5G relate to the historical concerns around EMF before discussing where the specific 5G conspiracy emerged from.

### 5G and EMF

One of the important takeaways from reading populist articles and books about 5G health concerns is that many activists do not believe that 5G is a completely novel threat. For example, books like *Radiation Nation* (Debaun and Debaun, 2017) and EMF*D (Mercola, 2020) stick fairly closely to existing arguments about EMF exposure outlined earlier in this article. To those authors, the distinct danger of 5G is that the networks will simply increase EMF exposure, exacerbating existing concerns. In fact, a book like EMF*D by Joseph Mercola reads almost like a second edition of an older book with a chapter or two on 5G thrown in. Mercola’s contention is that, at least in the short-term, 5G will not replace 4G networks. Rather, he asserts, 4G will remain operational and 5G transponders will boost speeds in more limited ranges on top of the 4G networks. Consequently, 5G will add to EMF exposure because the current exposure levels to 4G will not be reduced in the near future (Mercola, 2020: 668). In addition, anti-5G activists have also expressed health concerns around beamforming, which as noted earlier allows for a more focused signal to be broadcast to a specific device, and the higher frequencies at which 5G networks operate, despite the fact that these frequencies are still non-ionising and therefore safe.

While, at this point in time, there is, obviously, little research on the long-term effects of 5G, the research that does exist does not support the aforementioned health concerns. One of the largest studies was conducted by the International Commission on Non-Ionising Radiation Protection, which sets many radiation guidelines. They found that 5G networks exposed people to acceptable levels of radiation and there were no foreseeable risks. Thus, while there remain some outlier dissenting voices within the scientific community, the broad consensus opinion is that 5G is safe. Even so, several Swiss cantons have decreed a 5G moratorium until more research could be done (Jones, 2020; Seydtaghia, 2019), and multiple cities in the United States, along with Brussels in Belgium, have halted 5G rollouts in response to protests, health concerns or a combination of both (Mims, 2019; *The Brussels Times*, 2020).

It is also important to note that, alongside these more technical discussions, protesters and activists are also concerned with social transformations associated with 5G. Consultants and governments regularly suggest that 5G provides the necessary infrastructure to support advanced technologies, like autonomous vehicles and other technological developments that can help the vision of the ‘smart city’ become a reality. As such, protesters often note that 5G leads to more data centres, which can increase carbon emissions, argue that EMF can harm wildlife and connect 5G to broader concerns around technology and government surveillance (We say no to 5G in Australia, 2020b). These issues are not a core element of 5G activism but show that many in these groups are also generally disquieted with technological progress and often feel a sense of disempowerment in relation to government (We say no to 5G in Australia, 2020b). The expression of these concerns also points to many of the ongoing issues that the 5G rollout will have to overcome in the broader community, which may cause some strange intersections between activists concerned about EMF and others focused on the proliferation of small cells in urban spaces or other risks associated with smart city infrastructure.

## Section II: conspiracy

Having provided this context to 5G development and the longer history of concerns about the alleged deleterious effects of mobile and wireless technologies in the first part of the article, we now turn to the 5G-COVID-19 conspiracy itself. What is important to note here is that 5G-COVID-19 conspiracists are not operating in a vacuum and can be seen to draw inspiration from tried-and-tested ‘tactical repertoires of contention’ (Ogilvie and Rootes, 2016; Taylor, 2016) developed by past activists and protest movements, lobbyists and other conspiracists. These ‘repertoires’ include everything from the ‘selective perception and use of scientific knowledge’ (Weingart, Engels and Pansegrau, 2000: 274), various forms of direct action, intervention in policy-making at local, state and federal levels, and other forms of ‘coordinated subversion’ (Goldenberg, 2012), including organising in order to slow technology rollouts (which, as we will see, became much more direct and aggressive in connection with 5G infrastructure). Indeed, even the core concerns about electromagnetic sensitivity are not unique to mobile telecommunications. Anti-windfarm activists have also made similar erroneous claims about links between windfarms and electromagnetic sensitivity, and EMF exposure (McCallum et al., 2014).

### 5G and COVID-19

There are two types of conspiracy associated with 5G-COVID-19. One version suggests that radiation from 5G lowers your immune system, which makes you more susceptible to the virus (Shultz, 2020). The idea that some exposure may weaken your general immunity would appear to share some similarities with the earlier claim that sustained exposure to EMF could cause cancer. Indeed, this line of thinking was already present in anti-5G circles and it was merely amplified when the pandemic emerged as a new risk vector, one that directly challenged people’s immune system (Asher Hamilton, 2020a). The second and more prominent conspiracy moves well away from these historical concerns with EMF exposure. Instead, it argues that 5G directly causes COVID-19. There are a number of variations around this conspiracy theory. Among the more notable of them are the erroneous claims that (a) COVID-19 is a made up pandemic to cover up the deleterious effects of 5G radiation (Asher Hamilton, 2020a) and (b) that COVID-19 emerged from Wuhan because it had ‘been the guinea-pig city for 5G’ (Adams, 2020). Even more fantastical versions of this theory claim that the pandemic was ‘engineered by [. . .] Bill Gates, in an effort to depopulate an overcrowded planet’ (Davidson Sorkin, 2020; Wilson, 2020).

While it is hard to track where these conspiracies come from, it is clear that the existing 5G activist community provided some fertile ground for these theories. *Wired Magazine* identified a Belgian GP Kris Van Kerckhoven as a potential origin source (Temperton, 2020a). He claimed that 5G was life-threatening and that ‘it might be linked to coronavirus’ in an interview with a newspaper (Temperton, 2020a). This comment was ‘quickly picked up by anti-5G campaigners in the Dutch-speaking world’ and it started to circulate across activist communities on social media (Temperton, 2020a). This revelation sparked a new wave of content production among a range of disaffected groups from ‘New Agers [and] right-wingers [to] QAnon conspiracy theorists’, who want to share the ‘truth’ about the pandemic with the world (Broderick, 2020). However, a *BuzzFeed* investigation noted that many older videos, posted in the United Kingdom from mid-2019 onwards, when the 5G rollout started to occur there, were some of the more popular videos online (Broderick, 2020; see also, Waterson, 2020). These were often not directly related to the COVID-linked conspiracy but helped to stoke more general fears about 5G.

However, the conspiracy really hit the mainstream media when a number of notable celebrities started spreading the word. Woody Harrelson posted a video on Instagram that claimed ‘to show Chinese citizens toppling a 5G antenna’, and in another post referenced emeritus professor Martin Pall, who claimed that ‘Wuhan was China’s first “smart city” to incorporate the faster network’ (Shultz, 2020). Other notable spreaders of misinformation included rapper M.I.A., actor John Cusack (Gallagher, 2020), ‘boxer Amir Khan, singer Anne-Marie [. . .] former *Dancing on Ice* judge Jason Gardiner, pop star Keri Hilson, former *Made in Chelsea* star Lucy Watson, and TV personality Amanda Holden’ (Temperton, 2020a). All of these celebrities have large audiences on social media and this helped the conspiracy to move from the distant corners of the Internet to the mainstream (for detailed discussion, see Bruns, Harrington and Hurcombe, 2020). Explainers about the 5G conspiracy started appearing on news sites and health officials were increasingly asked about the issue, a phenomenon we discuss later in this article. It also placed increased attention on social media platforms and their handling of misinformation. While content moderation is not the focus of this article, it is worth noting that many platforms have placed information from the World Health Organization and Governments in prominent positions and have claimed to be focusing their misinformation efforts on COVID-19 (Adams, 2020).

### Conspiracy at the ground level: protests and arson

While many long-term activists may not necessarily agree with the conspiracy connecting COVID-19 to 5G, it may be that they viewed the renewed focus on 5G that came with these conspiracy theories as expedient to their overall anti-5G ambitions. At very least, the clear rise in protest activity during the pandemic suggests that committed 5G activists were at least partially energised by this increase in mainstream attention. In the United Kingdom, Australia and elsewhere, direct action was planned to stop the 5G rollout. This action appeared to be motivated by concern that the technology could harm the immune system, concerns that dovetail with older discourses around telecommunications and health risks. While many activists questioned claims that 5G could spread COVID-19, there appeared to be a general disdain about the risks caused by the virus given that regular protests were held during a time when most countries were enforcing social distancing and telling people to stay indoors.

Perhaps surprisingly, Australia became something of a global hotspot for these actions, many of which appear to be coordinated (or at least actively supported) by ‘We say no to 5G in Australia’ (2020a) and other associated groups (e.g. Northern Rivers for Safe Technology, 2020). The first protest occurred in late April when 100 people gathered to stop the installation of 5G infrastructure in Mullumbimby, a town in northern New South Wales. The main motivation for the protest was that 5G installation went ahead against council opposition, even though the installation was legal and did not require council authority (Bibby, 2020). Protesters blocked access to the site and had to be cleared by police (Bibby, 2020). Unlike celebrities’ social media posts mentioned earlier, no clear links were made to COVID-19. Indeed, ‘We say no to 5G in Australia’ (2020b, 2020c) criticised the media for making the link, arguing that ‘we were around long before CV!!’ However, the willingness to breach regulations and hold a large-scale gathering during a pandemic implied that they regarded the risks caused by 5G as of greater significance than coronavirus. It also underlined one of the recurrent concerns of anti-5G activists: that a greater density of small cells emitting radiation would be present around towns.

Another protest occurred in Melbourne, just as Australia was starting to open up again after the first wave of restrictions. Over 100 people stood in front of the Victorian parliament and railed against the lockdown laws. While the protest appeared to be similar to anti-lockdown demonstrations in the United States (Maqbool, 2020), there was no real focus on the economic impacts of the shutdown. Instead, the protest was a clearinghouse for conspiracy theorists, featuring ‘a colourful mix of coronavirus deniers, anti-vaxxers, 5G truthers, sovereign citizens, QAnon believers and other fringe conspiracy theorists’. Importantly for this article, alongside complaints about the new world order, protesters also voiced concerns about the tracking app that the Australian Government had released to assist with contact tracing and the broader 5G rollout (McGowan, 2020). While it is difficult to establish even tenuous causal connections between these issues, the protest highlighted some of the other foundational concerns animating concerns about 5G: it was largely sparked by restrictions stemming from the pandemic, and the protesters appeared to be less worried about the direct links between 5G and COVID-19 and more concerned with broad technological change and the spectre of totalitarian Government. As noted earlier, the 5G-COVID-19 conspiracy is often linked to a general feeling of disquiet with technological progress and a sense of disempowerment. So, in a sense, the protest showed how the 5G rollout and developments associated with COVID-19 both resonated with foundational concerns common to conspiracy theorists.

In contrast to Australia, where protestors have largely (if not solely) focused on public demonstrations, 5G-related protests in the United Kingdom have been more direct. There have been a spate of arson attacks on 5G towers across the country – with 77 towers set on fire between early April and early May 2020 (Asher Hamilton, 2020b), activity since repeated elsewhere, including in Australia – and people have also directed abuse at technicians installing mobile infrastructure (Tolhurst, 2020). This has involved ‘shouting and swearing at terrified key workers’, while other conspiracy theorists film these encounters and then upload them to social media (Temperton, 2020b). In one incident, an employee was spat on. There have also been a series of failed attempts to either set fire to other forms of mobile infrastructure or ‘damage [it] in other ways’ (Temperton, 2020b). Further examples of sabotage included putting anti-5G posters on infrastructure with razor blades and needles hidden behind them (BBC News, 2020). There is a clear link between these attacks and the conspiracy connecting 5G and COVID-19, as they only started occurring after the pandemic spread.

The conspiracy has been so prominent that it has forced its way into national briefings and associated public information. Downing Street in the United Kingdom has had to publicly dismiss the theory and speak to social media platforms about removing conspiracy-related content. In another example, the Australian Chief Medical Officer has directly addressed the growing spate of misinformation in a press conference (Murphy, 2020). State health departments have also started to actively disseminate information challenging the conspiracy, with New South Wales Health in Australia stating that ‘COVID-19 does not spread via mobile networks or wireless technology’ (NSW Health, 2020). While these public responses to the 5G-COVID-19 conspiracy attained a significant level of public prominence in early May 2020, conspiracy theorists have been involved for some time now in a sustained engagement with policy processes in order to challenge 5G rollout. As we explain below, these developments are of genuine concern to industry and government who are attempting to establish economic opportunities off the back of this infrastructural transformation.

### From protest to policy: disrupting 5G inquiries

While COVID-19 helped to focus mainstream attention on anti-5G campaigns, the pandemic conspiracy only provides a brief glimpse into the ongoing campaign to stop the rollout of 5G. This campaign forms part of a larger trajectory of long-standing health concerns around mobile telecommunications. As a result, the telecommunications sector and government are arguably more concerned about these more sustained efforts to disrupt the rollout rather than the pandemic-related conspiracy. Furthermore, these longer term attempts at rollout disruption have been relatively sophisticated. Activists have not just been disrupting installations and spreading misinformation on Facebook. They have also been strategically engaging with policy processes as councils and government start to assess the broader implications of the 5G rollout. While these inquiries are usually set up to examine potential economic opportunities and address concerns about the technology, they are often used by activists as sites to further advance their campaign agendas.

Perhaps the most notable example of this can be seen in the recent experience of the Glastonbury Town Council in the United Kingdom. They set up an inquiry in response to residents who regularly contacted council to let them know that they had ‘concerns about the safety’ of the technology and that it was ‘hazardous to human health and the environment due to the higher radiofrequency’ (Glastonbury Town Council, 2020: 4). However, the council mainly heard from ‘witnesses who had stated their support for a moratorium on the rollout of 5G’, including Martin Pall (Cellon-Jones, 2020). It was later revealed that an external member of the committee ‘was instrumental in choosing witnesses to appear before the committee’ and delivered ‘his own presentation in which he attacked the credibility of ICNIRP, the International Commission on Non-Ionizing Radiation Protection’ (Cellon-Jones, 2020). The council ended up not coming to a conclusion about the safety of 5G, but, in so doing, controversially suggested that there was some validity to the concerns of 5G activists. In reportage on the case, it was noted that the four volunteers on Glastonbury’s 5G Advisory Committee with ‘relevant expertise’ all resigned before the report was completed (Cellon-Jones, 2020). One of these volunteers, Mark Swain, who holds a physics degree, was quoted as saying, ‘I joined the working group in good faith, expecting to take part in a sensible discussion about 5G. Sadly the whole thing turned out to be a clueless pantomime driven by conspiracy theorists and sceptics’ (quoted in Cellon-Jones, 2020). While these local council resolutions cannot stop 5G rollout, they have played a role in solidifying community action against it. Indeed, a similar ban in Byron Shire helped to spark the protests in northern New South Wales, Australia. As noted earlier, the council tried to stop ‘development of the 5G tower until possible health and environmental risks were assessed by Telstra, the state and federal governments’ (Van Homrigh, 2020).

Co-ordinated action is also present at higher levels of government. The Australian House of Representatives Standing Committee on Communications and the Arts (2020) was directed to hold an inquiry into 5G in 2019. It was focused on understanding the ‘capability, capacity and deployment’ (p. vii) of the technology and its potential use-cases for government and businesses. However, the inquiry was flooded with concerns about 5G, requiring the committee to essentially go beyond its scope and directly address them. While there were the standard worries about EMF and health, people also submitted complaints about environmental harms and risks to individual privacy. The final report noted that ‘confidence in 5G has been shaken by extensive misinformation preying on the fears of the public spread via the internet’ (p. 41; see also, Taylor, 2020). The Australian Government has already launched ‘a [AUD]$9 million campaign to build public confidence in 5G’ (House of Representatives, 2020: 42), which will involve addressing misinformation, providing public education and supporting scientific research. After the experience of committee members, these efforts are likely to be extended with industry support. Both of these inquiries show that anti-5G campaigners are not just staying on the streets but are also attempting to engage with formal inquiries to advance their concerns.

### Economic possibilities and the importance of statecraft

Industry and policymakers are addressing these issues because they are motivated by a desire to realise the significant economic potential associated with 5G network technology. For instance, a 2017 IHS Markit report states that ‘the global 5G value chain will generate [US]$3.5 trillion in output’, enabling ‘[US]$12.3 trillion of global economic output, and supporting 22 million jobs, by 2035’ (IHS, 2017: 4). Figures very close to these appear in a 2020 white paper prepared by the World Economic Forum and Price Waterhouse Coopers (WEF-PwC, 2020). Similarly, a 2018 Deloitte report describes 5G as ‘fuel for economic growth’, suggesting that ‘5G will expand the network effect dramatically by extending the reach of the Internet to almost any kind of connection’ (Deloitte, 2018b: 2) and, in the process, generating a commensurate ‘data-network effect’ (*The Economist*, 2017). The Deloitte report, which is titled *5G: the chance to lead for a decade*, concludes that first mover advantage thus becomes vital, and that ‘countries that adopt 5G first [can be] expected to experience disproportionate gains in macroeconomic impact compared to those that lag’ (p. 2). The report notes that of those nations developing their 5G capacity, China is streets ahead of other countries, including the United States. ‘Since 2015’, the report states, ‘China outspent the United States by approximately [US]$24 billion in wireless infrastructure and built 350,000 new sites, while the United States built fewer than 30,000’ (Deloitte, 2018b: 1).

The expectations around 5G infrastructure are immense. The technology could allow ‘whole cities to be run with less moment-to-moment human intervention’ and help manage core operations from traffic management to urban lighting networks (Sanger et al., 2019). Establishing a baseline 5G infrastructure in key national locations will support economic efficiencies and will allow countries to take advantage of innovations that rely on this technology, like autonomous vehicles. As a result, there is growing competition between nation states to successfully build a network and limit the capacity of their rivals to develop one. This has manifested most clearly in a competition between the United States and China. These countries have vibrant national technology sectors that can exploit 5G technology and both are hoping that the rollout and subsequent innovation can provide a significant economic boost to the country. The risks to the United States in falling behind are stated in no uncertain terms in the Deloitte (2018b: 1) report: ‘China and other countries may be creating a 5G tsunami, making it near impossible to catch up’.

Deloitte’s analysis of the economic importance of 5G is noteworthy in that it points to the emergence of this technology as of growing geopolitical concern. This focus on geopolitics – and, more precisely, economic statecraft – provides a productive critical lens through which to view the current controversies swirling around 5G. According to William Norris, economic statecraft is defined as ‘the state’s intentional manipulation of economic interaction to capitalize on, reinforce, or reduce [. . .] associated externalities’ (Norris, 2016: 13–14), especially those that impact on economic stability and prosperity *and* national security. Norris coins the term ‘security externalities’ to capture this connection between economics and national security (pp. 12–13). Some of the examples cited as ‘types of security externalities arising from economic interaction’ include ‘sensitive technology transfer, loss of strategic industries, concentrated supply or demand dependence (in areas of trade, investment, and monetary relations)’ and so on. The second of these understandings of ‘security externalities’ as fuelling the ‘loss of strategic industries’ captures US concerns, as expressed by government representatives, consultants, strategists and journalists, about China’s 5G ambitions and Russia’s 5G-related disinformation campaigns.

The development of 5G network technology for international markets has become a three-way race between European, US and Chinese firms. With respect to the latter, Chinese telecom-equipment making firms have enthusiastically embraced China’s ‘outbound’, or ‘going out’ strategy, which Michael Keane and Haiqing Yu (2019: 4624) describe as ‘a term used by the Chinese government to rally the private sector (particularly platform capitalists) to internationalize’. These firms, such as ZTE and especially Huawei, have been at the centre of international tussles around control of 5G network infrastructure and national security concerns (see, for example, Bourke, 2020; Cave et al., 2018). In the US context, the Trump government appears to be viewing these tussles as primarily economic in nature. Some analysts, however, are more hawkish, regarding 5G rollout as a ‘geopolitical turning point’ and a national security issue of pressing importance (Gorman, 2020). While it remains unclear what role, if any, China has played in promulgating COVID-19-related 5G disinformation, COVID-19 has done little to slow its own rollout of 5G, which has continued apace (CGTN, 2020), or dampen China’s international ambitions regarding the supply of 5G infrastructure. On the latter, the US regards Huawei’s 5G push into Europe as ‘malign’ (Brennan, 2020b) and has expressed concern that China’s ‘mask diplomacy’ in Europe is itself being used as a mask to disguise their ongoing 5G ambitions in that market (Brennan, 2020a).

The Russian approach to economic statecraft has been somewhat different. This has involved, among other measures, pursuit of increasingly overt disinformation campaigns (Funke and Benkelman, 2019). While much of the attention on Russian disinformation campaigns to date has been focused on Russian troll farms and their interactions with and manipulations of social media accounts and platforms, increasing attention is being paid to their explicit use of more official channels, such as television broadcaster *RT* (Elliott, 2019). A report by the Policy Institute at Kings College London found that *RT* (and Russian state-controlled news agency, *Sputnik*) act as ‘negative news aggregators’, ‘harvesting, repackaging, and translating stories’ and producing content on European and North American democracies that is focused overwhelmingly on ‘political dysfunction, institutional failure, [and] social division’ (Ramsay and Robertshaw, 2019: 6). *RT* – which was once described as a ‘firehose of falsehood’ (Paul and Matthews, 2016) – has been accused of ‘giv[ing] the marginal a megaphone and traffic[king] in false equivalence’ (Broad, 2019). In its coverage of 5G, *RT* is said to have taken an ‘active role in stirring up apprehension’ around the health and other impacts of 5G network technology (Broad, 2019). It has also followed the same playbook in relation to COVID-19 (Broad, 2020). In relation to 5G, it has been said that *RT* is ‘less interested in serving the public than dulling Washington’s edge in the global race for the digital future’ (Broad, 2019).

How the US government responds to these moves – whether it continues to view China’s 5G interests as primarily an economic issue, or comes to hold the view that it is a ‘geopolitical turning point’ (Gorman, 2020), and what its longer term response is to persistent Russian disinformation campaigns – remains to be seen. For its part, the United Kingdom has reversed its decision to permit Huawei to be involved in its 5G networks, citing national security concerns (Shieber, 2020). Australia’s position is somewhat trickier. One view – expressed, for instance, by Australia’s previous prime minister, Malcolm Turnbull – is that Australia ought to develop infrastructure capability with its allies (Sadler, 2020). COVID-19 has intensified these concerns and has seen the members of the Five Eyes alliance work towards developing collective supply chains and working towards a stronger ‘economic dialogue’ (Benson, 2020). Given the online dominance of the United States and China, and the current instability in the United States, it may well be that Australia could be better placed engaging with other ‘middle powers’ and developing an alternative grouping. This is an unlikely to occur but establishing Australia’s own digital independence will be an ongoing issue for as long as the country is economically and strategically tied to these two powers.

## Conclusion

This article has examined conspiracy theories linking COVID-19 with 5G, with a particular focus on Australia, the United States and the United Kingdom. We began by providing a context to these theories, sketching a history of concern around mobile technologies and infrastructures, explaining what 5G technology is and what it entails, and examining concerns around EMF exposure and 5G. We then explored how conspiracies connecting COVID-19 to 5G played out on the ground, paying special attention to how conspiracists have engaged with government inquiries into 5G. In the final part of this article, we argued that a productive means of making fuller sense of what is happening with 5G is to look beyond these conspiracy theories to a set of larger, underlying and motivating geopolitical concerns, where the battle for control of 5G infrastructure can be understood as a form of economic statecraft.

Taking an international focus reveals the broader consequences of these conspiracy theories. It is not just people circulating false beliefs on social media. We have seen how individuals and groups attempt to influence policymaking processes and how countries can leverage these beliefs to disrupt the infrastructure rollouts of other countries. This is one key reason why nations are so concerned about the distribution of misinformation and disinformation. The circulation of uninformed or deliberately false information can not only seriously harm democratic outcomes but can also hinder economic development (Miller and Vaccari, 2020), as our study of 5G conspiracies shows. This is something that governments are evidently aware of and provides further explanation for the intensification of regulatory activity around misinformation and disinformation – consider, for example, the introduction of a voluntary code of practice on misinformation, which will be overseen by the Australian Communications and Media Authority (ACMA, 2020). The transformative potential of 5G is not something that governments can leave to chance and ensuring that progress is not delayed by the circulation of incorrect information is becoming a critical policy issue.

The notion of a global contest around 5G and the involvement of external nations in the circulation of disinformation also points to a wider geopolitical struggle around communications infrastructure. In closing, we suggest that this could present a new trajectory for communications research, one where the field engages more closely with international affairs. Indeed, recent Australian scholarship has addressed these geopolitical tensions either directly or indirectly. Wanning Sun and Haiqing Yu (2016), for example, have examined the use of Chinese social media platforms in Australia, and colleagues at Queensland University of Technology are starting to examine disinformation (Bruns, 2020; Bruns, Harrington and Hurcombe, 2020). This suggests that there is scope for the discipline to be more meaningfully involved in wider interdisciplinary discussions and public policy debates about how communications intersect with these international currents.

International analysts often use geoeconomics as a common alternative lens to think about relationships between nations, as it allows them to focus on how countries engage in the kind of economic statecraft described above. Considering the growing importance of communications in these geopolitical contests, *geocommunications* could be a productive conceptual term or frame for media and communication to explore in the future. This approach would be able to provide a fuller account of how nations have engaged with communications to realise their strategic goals. Our discussion of the infrastructural politics surrounding 5G provides just one example of how this broader term may be mobilised. Such an approach would also help the discipline engage productively with colleagues in international affairs who are increasingly interested in a range of topics that intersect with contemporary work occurring in the field.
